# High stearic acid diet modulates gut microbiota and aggravates acute graft-versus-host disease

**DOI:** 10.1038/s41392-021-00600-9

**Published:** 2021-07-23

**Authors:** Bingyu Yang, Xianfeng Zhang, Huanle Gong, Yuhui Huang, Chang Wang, Haiyan Liu, Chen Dong, Shoubao Ma, Xiaojin Wu, Depei Wu

**Affiliations:** 1 National Clinical Research Center for Hematologic Diseases, The First Affiliated Hospital of Soochow University, Jiangsu Institute of Hematology, Institute of Blood and Marrow Transplantation, Collaborative Innovation Center of Hematology, Soochow University, Suzhou, China; 2 Department of Hematology, The First Affiliated Hospital of Xi’an Jiaotong University, Xi’an, Shaanxi China; 3 Center for Clinical Laboratory, the First Affiliated Hospital of Soochow University, Suzhou, China; 4 Cyrus Tang Hematology Center, Collaborative Innovation Center of Hematology, the Key Laboratory of Stem Cells and Biomedical Materials of Jiangsu Province and the Chinese Ministry of Science and Technology, Soochow University, Suzhou, China; 5 School of Radiation Medicine and Protection, Medical College of Soochow University, Collaborative Innovation Center of Radiological Medicine of Jiangsu Higher Education Institutions, Jiangsu Provincial Key Laboratory of Radiation Medicine and Protection, Suzhou, China; 6 Immunology Programme, Life Sciences Institute and Department of Microbiology and Immunology, Yong Loo Lin School of Medicine, National University of Singapore, Singapore, Singapore; 7 Institute for Immunology and School of Medicine, Tsinghua University, Beijing, China

**Keywords:** Haematological cancer, Immunological disorders

**Dear Editor,**

Acute graft-versus-host disease (aGVHD) is the leading cause of transplantation-related mortality, and limits therapeutic benefits of allogeneic bone marrow transplantation (allo-BMT). New insight is needed into the development of aGVHD. Most nutritional metabolites contribute to host health and immune homeostasis. We previously demonstrated that serum levels of stearic acid (SA) and palmitic acid (PA), the most abundant long-chain saturated fatty acids in the human body, were reliable biomarkers for predicting aGVHD in patients after allo-BMT,^1^ suggesting these two metabolites were likely involved in the pathogenesis of aGVHD, although the mechanisms remained unclear.

In an established aGVHD mouse model, we fed recipient mice with either high stearic acid diet (HSAD) or high palmitic acid diet (HPAD) to evaluate the effects of PA or SA intake. Administration of HPAD showed no deleterious impact on aGVHD (Supplementary Fig. 1a–c). By contrast, HSAD recipients exhibited significantly increased aGVHD mortality, more severe pathological damage, reduced body weight, higher aGVHD clinical scores, and elevated levels of most serum lipids compared with normal diet (ND) recipients after allo-BMT (Fig. 1a, b and Supplementary Fig. 1d–f). These results were consistent with HSAD recipients from another aGVHD model; however, supplementation of SA in donors did not affect mortality rates (Supplementary Fig. 2a–f). These data indicate that elevated SA could exacerbate aGVHD severity and mortality.

**Fig. 1:**
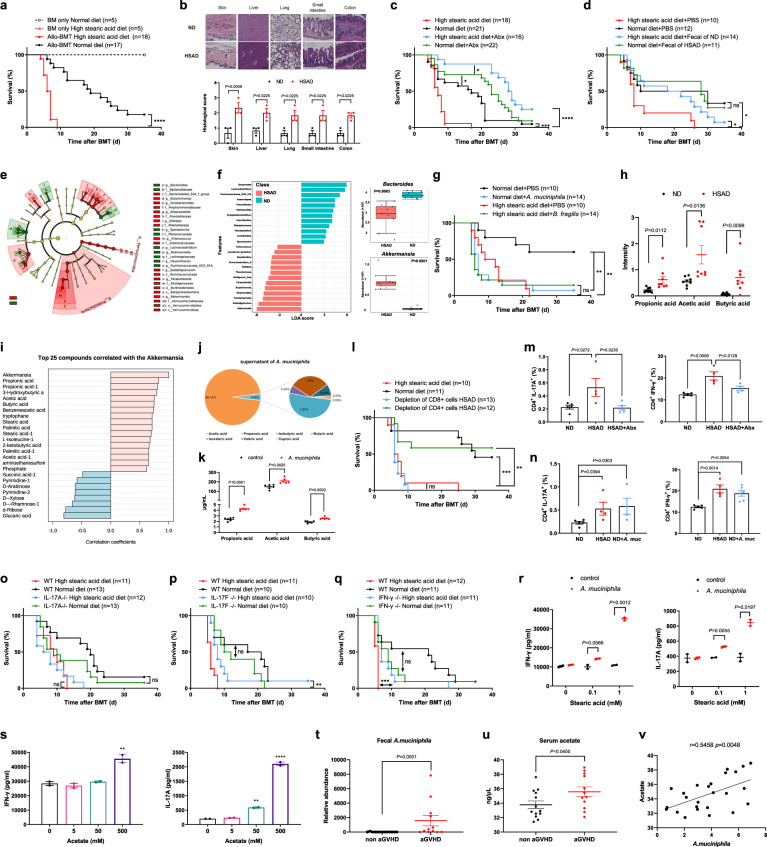
High stearic acid diet (HSAD) modulates gut microbiota and aggravates acute graft-versus-host disease (aGVHD). **a** Survival of BALB/c recipient mice treated with normal diet (ND) or high stearic acid diet (HSAD) received bone marrow transplantation (BMT) from either syngeneic or allogeneic C57BL/6 donors. **b** Representative images and histopathological aGVHD scores for each aGVHD target organ on day 7 after allo-BMT, including skin, liver, lung, small intestine, and colon. Scale bars, 100 µm. **c** Survival of ND and HSAD recipients after allo-BMT treated with antibiotics (Abx) in drinking water. **d** Survival of ND and HSAD recipients after allo-BMT treated with fecal microbiota transplantation. **e** Taxonomic cladogram obtained from linear discriminant analysis effect size (LEfSe) showing bacterial taxa (phylum, class, and order) that were differentially abundant in HSAD and ND mice. Red indicates increased abundance in HSAD mice; green indicates increased abundance in ND mice. **f** Results of LEfSe analysis showing bacterial genus that were significantly different in abundance between HSAD and ND mice. **g** Survival after allo-BMT of ND mice receiving *A. muciniphila* and HSAD mice receiving *B. fragilis* compared to PBS vehicle. **h** Relative abundance of propionic acid, acetic acid, and butyric acid in feces of ND and HSAD mice (*n* = 8 per group). **i** Correlations of *A. muciniphila* levels with of stool metabolite levels, as determined by Spearman’s rank test. Red columns indicate significant positive correlations (Spearman’s correlation value *r* > 0.6, adjusted *p* < 0.05), and blue columns indicate significant negative correlations (*r* < −0.5, *p* < 0.05), except for succinic acid (*p* > 0.05). **j** Pie chart of seven representative SCFAs from bacterial cultures of *A. muciniphila*, each color represents one SCFA. **k** Levels of propionic acid, acetic acid, and butyric acid in bacterial culture supernatant and no-bacteria control. For **j** and **k**, *n* = 6 per group. **l** Survival after allo-BMT of HSAD recipients receiving donor cells in the absence of CD4^+^ T cells or CD8^+^ T cells. **m** Quantification of IL-17A^+^ Th17 and IFNγ^+^ Th1 lymphocytes in ND, HSAD, and antibiotic-treated HSAD (HSAD-Abx) recipients (*n* = 4–5 per group). **n** Quantification of IL-17A^+^ Th17 and IFNγ^+^ Th1 lymphocytes in ND mice, HSAD and ND mice receiving *A. muciniphila* (ND + *A. muciniphila*) recipients (*n* = 4–5 per group). **o**–**q** Lethally irradiated BALB/c ND and HSAD recipients were treated with WT B6 donors and IL-17A^−/−^ B6 donors, IL-17F^−/−^ B6 donors, and IFN-γ^−/−^ B6 donors. Survival time was monitored. **r** Levels of IL-17A and IFN-γ were analyzed by ELISA between *A. muciniphila* supernatant and control co-cultured with stearic acid medium with different concentrations (0, 0.1, and 1 mM) under Th17- or Th1-stimulating conditions. **s** Secretion of IL-17A and IFN-γ from the supernatant of cultured naive CD4^+^ T cells under Th17- or Th1-stimulating conditions treated with various concentrations of acetate. For **r** and **s**, *n* = 2 per dose. **t**–**u** Quantification of *A. muciniphila* from the feces, and levels of acetate in the serum of non-aGVHD subjects (*n* = 13) and individuals with aGVHD (*n* = 12). **v** Correlations between the concentration of *A. muciniphila* in fecal samples and acetate in serum samples, as determined by Pearson’s rank test. Survival curves were compared using a log-rank (Mantel–Cox) statistical test. Comparisons between two groups were assessed using a two-tailed Student’s *t* test or Mann–Whitney *U* test. Multiple comparisons were evaluated statistically by two-way ANOVA, one-way ANOVA, or Kruskal–Wallis. Data are presented as the mean ± SEM. Exact *p* values are reported or presented as *p < 0.05, **p < 0.01, ***p < 0.001, or ****p < 0.0001

Since SA is an essential dietary component, and dietary nutrients can drive gut microbial community structure, we hypothesized that gut microbiota participated in promoting SA function. We found that antibiotic-induced microbiome depletion reduced mortality of both HSAD and ND recipients (Fig. 1c and Supplementary Fig. 3a, b). Moreover, fecal microbiota transplantation from ND to HSAD recipients attenuated the exacerbated aGVHD phenotype of HSAD mice (Fig. 1d and Supplementary Fig. 3c, d). Collectively, these results suggested that gut microbiota contribute to the development of HSAD-mediated severe aGVHD.

Next, we explored the microbiome profiles of HSAD and ND recipients. 16S ribosomal DNA sequencing showed significantly increased microbial diversity in HSAD mice, potentially through the loss of commensal bacteria and relative increase in nondominant species (Supplementary Fig. 3e). Principal component analysis revealed different microbial community structures between HSAD and ND recipients (Supplementary Fig. 3f). Indeed, linear discriminant analysis effect size (LEfSe) method showed distinct microbial profiles in both groups (Fig. 1e). Interestingly, *Akkermansia* was among the dramatically enriched genera in HSAD samples, in agreement with other studies using high-fat diet mouse models,^2^ while genus *Bacteroides* showed the largest increase in abundance among ND samples (Fig. 1f).

These in vivo results suggested that gut microbiome depletion led to the reduced aGVHD mortality of HSAD recipients, whereas administration of *Akkermansia muciniphila* aggravated aGVHD mortality of ND recipients, which was consistent with previous reports.^3^ However, *Bacteroides fragilis* administration did not improve the survival of HSAD mice (Fig. 1g and Supplementary Fig. 4a, b). Effective gut colonization was validated by markedly higher levels of *A. muciniphila* in feces of colonized ND mice, compared to PBS-treated controls (Supplementary Fig. 4c, d). These data indicate that the accumulation of *A. muciniphila* in HSAD recipient mice can result in exacerbated aGVHD.

We then profiled the fecal metabolomes of HSAD and ND mice, which revealed distinct separation of metabolic patterns between groups. Heatmap analysis indicated several significantly differential metabolites between dietary groups, especially enrichment for short-chain fatty acids (SCFAs) in HSAD mice (Supplementary Fig. 5a, b). Notably, the significant increases in acetic acid (acetate), butyric acid (butyrate), and propionic acid (propionate) in HSAD mice (the three highest-abundance SCFAs in intestinal lumen) were positively correlated with *A. muciniphila* levels (Fig. 1h, i). We determined the concentrations of seven representative SCFAs from *A. muciniphila* supernatant and found that consistent with our in vivo data, most SCFA concentrations were significantly upregulated compared with no-bacteria controls, with acetate showing the highest abundance (Fig. 1j, k). High-dose acetate administration also exacerbated aGVHD in ND mice (Supplementary Fig. 5c, d). These data indicate that *A. muciniphila* may modulate aGVHD by altering SCFA metabolism, especially acetate.

Autoreactive T cells, including CD4^+^ and CD8^+^ T cells, have been proposed to contribute to aGVHD. We characterized a robust increase in activated, effector, and memory CD4^+^ T cells in the spleens of HSAD mice on day 7 after allo-BMT (Supplementary Fig. 6a, b). We also noted reduced mortality rates in recipients of donor cells in the absence of CD4^+^ T cells, but not CD8^+^ T cells, indicating an essential role of CD4^+^ T cells in HSAD-mediated severe aGVHD (Fig. 1l and Supplementary Fig. 7a, b). Further T cell polarization analyses revealed that HSAD mice developed a strongly skewed Th17 and Th1 response, characterized by high frequencies of IL-17A^+^ and IFN-γ^+^ cells within CD4^+^ T lymphocytes. Moreover, antibiotic treatment could effectively reduce pro-inflammatory Th1 and Th17 cells in HSAD mice, whereas *A. muciniphila*-colonized ND mice had significantly increased proportions of these cells (Fig. 1m, n and Supplementary Fig. 7c, d).

Among immune cell-produced cytokines, we identified higher serum levels of IFN-γ, IL-17A, and IL-17F in HSAD recipients versus ND recipients (Supplementary Fig. 7e). Supporting this finding, we observed significant attenuation of aGVHD in HSAD recipients transplanted from IL-17F^−/−^ and IFN-γ^−/−^ donors versus WT donors. However, blocking IL-17A by using IL-17A^−/−^ donors did not rescue lethality (Fig. 1o–q and Supplementary Fig. 8a–c), suggesting that HSAD aggravation of aGVHD is mainly mediated by IL-17F and IFN-γ, but not IL-17A. Taken together, these results show that enhanced Th17 and Th1 responses and downstream cytokines contribute to HSAD-mediated exacerbation of aGVHD.

To test the effects of *A. muciniphila* on T cells in vitro, we firstly demonstrated that SA promoted growth of *A. muciniphila* (Supplementary Fig. 9a). We then exposed naive CD4^+^ T cells to *A. muciniphila* extracts or no-bacteria control under pathogenic Th17- or Th1-stimulating conditions. Cells co-treated with SA exhibited higher levels of IL-17A and IFN-γ secretion compared to the no-SA control. Moreover, bacteria-treated cells exhibited significantly increased proportions of Th17 and Th1 cells compared to controls (Fig. 1r and Supplementary Fig. 9b). In addition, high concentrations of acetate, comparable to that in *A. muciniphila* supernatants, also promoted the differentiation of Th17 and Th1 cells (Fig. 1s and Supplementary Fig. 9c).

Finally, we explored the relevance of these findings to aGVHD outcomes in human patients. A total of 25 recipients of allo-BMT transplants at our center were enrolled (Table S1). Consistent with our findings in mice, aGVHD patients had significantly higher concentrations of *A. muciniphila*, acetate, IL-17A, and IFN-γ (Fig. 1t, u) compared to non-aGVHD patients. Positive correlations were also observed between *A. muciniphila* and acetate, and between *A. muciniphila* and IL-17A or IFN-γ in patients (Fig. 1v and Supplementary Fig. 10). Previous studies have established that *A. muciniphila* and SCFAs mediate beneficial effects. However, *A. muciniphila* has also been reported to contribute to inflammation during infection, and in mice with normal gut microbiota.^4^ Although the SCFA butyrate has been shown to mitigate GVHD, acetate administration provided no benefit to aGVHD.^5^ Moreover, exposure to high acetic acid doses can induce colonic inflammation and has therefore been used to establish colitis models. In the context of intense systemic injury and neutropenia after allo-BMT, it is difficult to definitively determine which bacteria and metabolites confer purely beneficial or harmful effects on aGVHD.

Here, we provide the first evidence that HSAD aggravates aGVHD through enrichment of *A. muciniphila* and its metabolite, acetate. These findings suggest that the modulation of gut microbiota and associated metabolites may represent new therapeutic targets for the prophylaxis and treatment of aGVHD.

## Supplementary information

Supplementary Materials

## Data Availability

All data generated or analyzed during this study are available within the article and its supplementary files or from the corresponding author upon reasonable request.
