# Supplementary material for: High stearic acid diet modulates gut microbiota and aggravates acute graft-versus-host disease
Source: Signal Transduct Target Ther. 2021 Jul 23;6:277. doi: 10.1038/s41392-021-00600-9 (PMC8298483; doi:10.1038/s41392-021-00600-9)
Supplement: Supplementary file 1 — Supplementary Materials [file 41392_2021_600_MOESM1_ESM.docx]

Supplementary Materials for

High stearic acid diet modulates gut microbiota and aggravates acute graft-versus-host disease

Bingyu Yang ^1,2#^, Xianfeng Zhang ^3#^, Huanle Gong ^1#^, Yuhui Huang ^4^, Chang Wang ^5^, Haiyan Liu^6^, Chen Dong ^7^, Shoubao Ma ^1*^, Xiaojin Wu ^1*^, Depei Wu ^1*^

Correspondence to: [wudepei@suda.edu.cn](mailto:xxxxx@xxxx.xxx)

**This PDF file includes:**

Materials and Methods

References

Figures. S1 to S10

Tables S1

Materials and Methods

Human fecal and serum samples

The study was approved by the Faculty Hospital Ethics Committee at the First Affiliated Hospital of Soochow University in accordance with the guidelines in the Declaration of Helsinki (Document no. 2017-289). All subjects provided written informed consent from individuals or guardian participants before the start of this study. Patients analyzed in this study were all hospitalized for allogeneic hematopoietic stem cell transplantation in the First Affiliated Hospital of Soochow University, Jiangsu, China. We recruited 25 allo-BMT recipients, 12 individuals with aGVHD and 13 individuals as non-aGVHD control. Clinical characteristics were extracted from patient medical records and detailed in Table S1. All patients received the same antibiotic regimen to complete gut decontamination before allo-BMT. Stool samples were collected during aGVHD or non-aGVHD. All the serum samples from day 7 after allo-BMT were obtained from Jiangsu Biobank of Clinical Resources.

**Mice and diet**

Female C57BL/6 (H-2^b^) and BALB/c (H-2^d^) mice of 5-7 weeks old and weighing 18-21 g were purchased from Shanghai SLAC Laboratory Animal Co., Ltd (Shanghai, China). C57BL/6 IL-17A^-/-^ and C57BL/6 IL-17F^-/-^ mice were kindly provided by Dr. Chen Dong from Tsinghua University. C57BL/6 IFNγ^-/-^ mice were obtained from Dr. Zhinan Yin from Jinan University and Dr. Yuhui Huang from Soochow University.

After 1-2 weeks of acclimation on a normal chow diet, mice were randomly assigned to normal diet (ND) or high stearic acid diet (HSAD) or high palmitic acid diet (HPAD). The composition of the diet for mice were detailed in a previous study^1^ and were provided by Double Lion Experimental Animal Feed Technology Co., Ltd. (Suzhou, China). Mice were adapted to ND/HSAD/HPAD diet for 4 weeks prior to allo-BMT were performed.

All the mice were bred and maintained in a specific pathogen-free barrier facility under 20-25°C with standard 12:12 h light/dark conditions and were co-caged at less than five mice per cage at Soochow University. Animals were given unrestricted access to food and water. Each animal experiment was carried out independently at least twice. All animal experiments were performed in accordance with the National Animal Care and Use Committee after approval by the Institutional Laboratory Animal Care and Use Committee of Soochow University (Suzhou, China).

**Animal Treatments**

aGVHD model

Allogeneic (C57BL/6 to BALB/c) murine aGVHD model was induced as previously described.^2^ In brief, the wild type (WT) C57BL/6 female recipients were received lethal total body irradiation (800 cGy, one dose), and WT BALB/c female recipients were received lethal total body irradiation (650 cGy, one dose) from X-ray, then 1 × 10 ^7^ bone marrow cells and 5 × 10 ^6^ splenocytes of allogeneic donors was injected via the tail vein on day 0. The clinical severity of aGVHD was evaluated every other day from day 3 to day 35 after allo-BMT according to a clinical aGVHD scoring system as described.^3^ CD4^+^ and CD8^+^ T cell depletion from donor mice spleen T cells were performed by EasySep™ Mouse CD4 Positive Selection Kit II and EasySep™ Mouse CD8^+^ T Cell Isolation Kit (StemCell Technologies, Vancouver, BC, Canada) according to the manufacturer’s protocols.

Fecal microbiota transplantation

For fecal microbiota transplantation, stool samples were collected from four individuals with HSAD or ND for 4 weeks. At least 5 pellets of fresh feces were collected from each mouse in the HSAD and ND groups, transferred into sterile conical tubes, pooled, vortexed and re-suspended in sterile filtered autoclaved PBS and adjusted to a final volume of 200 mg feces/ml. Then the fecal suspension was applied by oral gavage (in 200 µL PBS) in order to reconstitute mice with a complex intestinal microbiota (ND+fecal of HSAD and HSAD+ fecal of ND). PBS was used (ND+PBS and HSAD+PBS) as control. Each recipient mouse received 200 µL of fecal suspension or PBS every other day. No antibiotic treatment was involved in the process.

Antibiotic treatment

HSAD and ND mice were depleted of all detectable commensal bacterial by a cocktail of antibiotic 1 ^4^ via autoclaved drinking water (ND+Abx and HSAD+Abx) for 4 weeks prior to allo-BMT. After allo-BMT, to maintain the intestinal depletion, mice were subsequently switched over to a different antibiotic cocktail 2 ^5^ for 4 weeks. Control mice were given normal drinking water at the same time (ND-c and HSAD-c). Antibiotic cocktail 1 contains 1 g/L each of ampicillin, metronidazole and neomycin and 0.5 g/L of vancomycin. Antibiotic cocktail 2 contains 2 g/L of streptomycin, 0.17 g/L of gentamicin, 0.125 mg/L of ciprofloxacin, and 1 g/L of bacitracin. Among all the antibiotics mentioned above, bacitracin was purchased from Sinopharm Chemical Reagent Co., Ltd., (Shanghai, China), gentamicin was purchased from Sangon Biotech Co., Ltd., (Shanghai, China) and others were purchased from BBI Life Science Co., Ltd. (Shanghai, China).

Acetate treatment

HSAD and ND mice received vehicle (200 µL PBS), and ND mice received acetate (100 mg kg^−1^ per day, Sigma Aldrich, St. Louis, MO, USA) by intraperitoneal injection for 1 week and every other day thereafter from day -21 to day +7.

**Detection of serum lipids, PA and SA**

For serum lipids, blood samples were collected at 0, 1, 2, 3 and 4 weeks after administration of ND or HSAD, and serum was stored at -80°C until analysis. Serum T-CHO, TG, LDL cholesterol (LDL-c) and HDL cholesterol (HDL-c) were measured using kits purchased from Nanjing Jiancheng Bioengineering Institute (Nanjing, China).

**Bacteria culture and administration to mice**

*A. muciniphila* (BAA-835, American Type Culture Collection, Manassas, VA) were cultured in BHI (brain-heart-infusion) broth (BD Biosciences, San Jose, CA, USA) or on Columbia Blood Agar (Autobio Diagnostics Co., Ltd, Zhengzhou, China) at 37°C in an anaerobic chamber. *B. fragilis* strain 25285 was also purchased from ATCC and cultured in anaerobically sterilized Trypticase soy broth (BD Biosciences, San Jose, CA, USA) or on Columbia Blood Agar at 37°C. For maintaining the anaerobic conditions, GENbag anaerobic systems (Biomeriux, France) were used. To verify the effect of stearic acid (Sigma-Aldrich, St. Louis, MO, USA) on the growth of *A. muciniphila*, SA dissolved in ethanol (Sinopharm Chemical Reagent Co., Ltd., Shanghai, China) was then added into a tube containing BHI liquid media and grown at 37°C anaerobically for 3 days. The concentration of bacteria was calculated by measuring the absorbance at the wavelength of 600 nm.

To study the influence of *A. muciniphila* and *B. fragilis* supplementation, they were scraped from the agar plates and diluted in sterile PBS. Cultures were centrifuged at 2000 g for 10 min, washed with sterile PBS once and re-suspended in sterile PBS. The ND mice were treated with *A. muciniphila* by oral gavage at a dose of 1.5×10 ^9^ cfu /200 µL suspended in sterile anaerobic PBS (ND+ *A. muciniphila*). Similarly, 5×10 ^8^ cfu of *B. fragilis* in 200 µL sterile anaerobic PBS was orally gavaged into HSAD mice (HSAD+ *B. fragilis*). As control, an equivalent volume of sterile anaerobic PBS was orally administered (ND+PBS and HSAD+PBS). Treatments performed every other day and were continued for 4 weeks.

**ELISA**

For serum cytokines, blood samples were obtained from surviving recipient mice on day 6 after BMT and stored at -80°C. Levels of mouse IL-17A, IL-17F, and interferon gamma (IFN-γ) were examined by a bead-based multiplex assay panel according to the instructions provided by the manufacturer (BioLegend, San Diego, CA, USA). The concentrations of IL-17A and IFN-γ in culture supernatant were measured using commercial ELISA kits from (Dakewe Biotech Co., Ltd., Shenzhen, China) according to the manufacturer’s instruction. All blood samples and cell culture supernatant were centrifuged at low speed and stored at -80°C.

**Cell preparation and flow cytometry**

Splenocytes were obtained from mice at day 5-7 after BMT. Procedures of isolating single cell suspensions from spleen were performed according to our previous study.^6^ For cell surface staining, cell samples were labeled with conjugated mAb for 20 min at 4°C in the presence of purified anti-CD16/32 (eBioscience, San Diego, CA, USA) at saturation to block unspecific staining.

For intracellular cytokine staining, cells were stimulated for 5 h with phorbol-12-myristate-13-acetate (PMA, 50 ng/ml) and ionomycin (500 ng/ml) in the presence of brefeldin A (10 µg/ml) or with Leukocyte Activation Cocktail with GolgiPlug (BD Pharmingen, San Diego, CA, USA) at 37°C with 5% CO2. Following the stimulation, cells were fixed using CytoFix/CytoPerm buffer (BD Biosciences, San Jose, CA, USA) and stained with antibodies against intracellular cytokines or isotype control on ice for 30 min.

The following antibodies were used for staining: FITC-anti-mouse-CD69 (H1.2F3), PE/CF594-anti-mouse-CD3 (145-2C11), PerCP/Cy5.5-anti-mouse-CD4 (RM4-5), and APC/H7-anti-mouse-CD4 (GK1.5) were purchased from BD Biosciences (San Jose, CA, USA). The FITC-anti-mouse-CD8 (53-6.7), FITC-anti-mouse-CD4 (GK1.5), PE-anti-mouse-IL-17A (TC11-18H10.1), PE-anti-mouse-IFN-γ (XMG1.2), PE-anti-mouse-H-2Kb (AF6–88.5), APC -anti-mouse-CD4 (RM4-5), APC-anti-mouse-CD8 (53-6.7), Alexa fluor 700-anti-mouse-CD45 (30-F11), and APC-anti-mouse-IFN-γ (XMG1.2) were purchased from BioLegend (San Diego, CA, USA).

After gating for lymphocytes and excluding doublets, living cells were included in further analyses. Unstained and single color were used to identify stained populations. Cells were acquired on a NovoCyte Flow cytometer (ACEA Biosciences, San Diego, CA, USA) and analyzed using Flowjo software (FlowJo, Ashland, OR, USA).

***A. muciniphila* extracts preparation**

*A. muciniphila* extracts were prepared as previously reported ^7,8^ with a few modifications. All T cell responses to *A. muciniphila* were compared to no-bacteria vehicle controls but with stearic acid (Sigma-Aldrich, St. Louis, MO, USA) of 0, 0.1 and 1 mM (con). *A. muciniphila* was adjusted to 1 x 10^8^ /ml and was grown on BHI broth in the presence of SA with the same concentration gradient compared to vehicle controls. *A. muciniphila* and vehicle controls were grown at 37°C in an anaerobic chamber. After 3 days, the supernatant and pellets were obtained by centrifugation at 6000 rpm at 4°C for 10 min. The isolated suspensions were passed through 0.22-µm cell strainers, heat-inactivated at 65°C for 1 h, sonicated for 10 min and were collected as *A. muciniphila* extracts (*A. muc*).

**Murine T cell differentiation**

Splenic T cells of female C57BL/6 mice were isolated and naive CD4^+^ T cells were sorted by magnetic­activated cell sorting using EasySep™ Mouse Naïve CD4^+^ T Cell Isolation Kit (StemCell Technologies, Vancouver, BC, Canada) according to the manufacturer’s instructions. Naive CD4^+^ cells were plated at 1 x 10^5^ /well into wells precoated with anti-mouse CD3ε, clone 145-2C11 (5µg/ml) and anti-mouse CD28, clone 37.51 (2µg/mL). For Th17 cell differentiation, sorted naive CD4^+^ T cells were stimulated by IL-6 (50 ng/ml), IL-23 (10 ng/ml), IL-1β (10 ng/ml), rhTGF-β1 (2.5 ng/ml), anti-IL-4 (10 µg /ml) and anti-IFN-γ (10 µg /ml) for 3 days. For Th1 cell differentiation, naive CD4^+^ T cells were cultured for 3 days with IL-2 (100UI /ml), IL-12 (10 ng/ml) and anti-IL-4 (10 µg /ml). All the antibodies mentioned above were purchased from BioLegend (San Diego, CA, USA).

To verify the influence of *A. muciniphila* on T cell differentiation, cells were cultured with vehicle (BHI+SA) or *A. muciniphila* under different treatment for 3 days at the same conditions. For SCFAs treatment, cells were treated with sodium acetate (Sigma Aldrich, St. Louis, MO, USA) *in vitro*.

**Histopathologic analysis**

For histopathologic analysis, representative samples of liver, lung, small intestine, colon and skin were obtained from transplanted recipients at day 7 after BMT and tissues were fixed in 10% neutral-buffered formalin. Samples were then dehydrated, embedded in paraffin, sectioned and stained with hematoxylin-eosin according to the standard protocol. Tissue damage was analyzed for pathologic scoring by a semiquantitative scoring system as previously described.^9,10^

**Fecal sample collection and microbiome analysis**

Fresh fecal samples from HSAD and ND mice were obtained at day 0 of allo-BMT. Each sample was divided into two parts: one part was used for analysis of the gut microbiota and the other was used for metabolomics. All fecal samples were immediately frozen, stored at -80°C and analyzed within 2 months.

Microbial genomic DNA was extracted from each fecal sample (0.1 g) using cador Pathogen 96 QIAcube HT Kit (QIAGEN, Duesseldorf, Germany). Next generation sequencing library preparations and Illumina MiSeq sequencing were conducted at GENEWIZ, Inc. (Suzhou, China). DNA samples were quantified using a Qubit 2.0 Fluorometer (Invitrogen, Carlsbad, CA, USA). 30-50 ng DNA was used to generate amplicons using a MetaVx™ Library Preparation kit (GENEWIZ, Inc., South Plainfield, NJ, USA). The v3 and v4 regions were amplified using forward primers containing the sequence “CCTACGGRRBGCASCAGKVRVGAAT” and reverse primers containing the sequence “GGACTACNVGGGTWTCTAATCC”. The QIIME data analysis package was used for 16S rDNA data analysis. Sequences were grouped into operational taxonomic units (OTUs) using the clustering program VSEARCH (1.9.6) against the Silva 119 database pre-clustered at 97% sequence identity. Taxonomic annotation was conducted using a ribosomal database project (RDP) classifier. Based on the results of the OTU analysis, alpha and beta diversity analyses, principal coordinate analysis (PCoA) and linear discriminant analysis effect size (LEFse) analysis were performed for all samples, to provide information about species diversity, clustering, and composition differences in community structures. Data processing included the use of MetaboAnalyst (https://www.metaboanalyst.ca/).

**Confirmation of *A. muciniphila* and *B. fragilis* colonization**

To verify that mice were colonized with *A. muciniphila* and *B. fragilis*, their relative abundance was examined by a 16S rDNA quantitative PCR (qPCR) approach using SYBR Green Master Mix (Applied Biosystems, Warrington, UK). The primers used to detect bacteria were based on 16S rDNA gene sequences: *A. muciniphila*, forward CAGCACGTGAAGGTGGGGAC, reverse CCTTGCGGTTGGCTTCAGAT; *B. fragilis*, forward CTGAACCAGCCAAGTAGCG, reverse CCGCAAACTTTCACAACTGACTTA. Differences were calculated using the comparative 2^-ΔΔCt^ method. Results obtained were normalized to those for the 16S rDNA gene (the V3 and V4 regions, detailed above) and compared with the mean target gene expression.

**Metabolome profiling of fecal samples**

Eighteen fecal samples (9 ND and 9 HSAD) were prepared for gas chromatography-mass spectrometry (GC-MS) metabolomics analysis. Briefly, a 100-mg fecal sample was mixed with 500 μL methanol and then 20 mg/mL 2,4-dichlorobenzoic acid (internal standard) was added. After vortexing and centrifugation, the supernatant was collected. Full details for drying with vacuum and derivatization referred to our previous report.^11^ Metabolic profiling of fecal samples was acquired using an TSQ 8000 Evo GC-MS (Thermo Fisher Scientific, Waltham, MA, USA). Each derivatized sample (1 μL) was injected into a DB-5 fused silica capillary column (30 m × 0.25 mm × 0.25 μm, J&W Scientific, Folsom, CA, USA) with a split ratio of 100:1. The response area of each metabolite was finally normalized to internal standard 2,4-dichlorobenzoic acid (Sigma-Aldrich, St. Louis, MO, USA). GC-MS raw data were acquired from XCalibur software version 2.2 (Thermo Fisher Scientific, Waltham, MA, USA) and details of further analysis have been reported.^11^

**Short-chain fatty acids analyses**

GC-MS-based targeted seven representative short-chain fatty acids (acetic acid, propionic acid, butyric acid, valeric acid, caproic acid, isobutyric acid and isovaleric acid) and measurements were conducted at BioNovoGene Co., Ltd. (Suzhou, China). In brief, samples were thawed and 100 μL aliquots were mixed with 50 μL 15% phosphoric acid. Subsequently, 10 μL internal standard (isohexanoic acid，75 μg/mL) and 140 μL ether were added. After vortexing for 30 s, the sample was centrifuged for 10 min to precipitate the protein (12,000 rpm at 4°C). The supernatant was transferred into a new tube and then analyzed by GC-MS. All samples, including standard Free Fatty Acids (FFAs), were analyzed using a HP-INNOWAX capillary column (30 m × 0.25 mm × 0.25 μm, J&W Scientific, Folsom, CA, USA) on a Thermo TRACE 1310-ISQ LT GC/MS system (Thermo Fisher Scientific, Waltham, MA, USA). Peaks representing each metabolite were extracted and analyzed according to standard FFAs peaks.

**Statistical Analysis**

Statistical analysis was performed with GraphPad 8.0 Prism (GraphPad Software Inc., La Jolla, CA, USA). Survival curves were compared using a Log-Rank (Mantel-Cox) statistical test. Comparisons between two groups were assessed using a two-tailed Student t test or Mann-Whitney U test after checking for normal distribution. Multiple comparisons were evaluated statistically by one-way ANOVA or Kruskal-Wallis. Data are presented as mean ± SEM; **p* < 0.05, ***p* < 0.01, ****p* < 0.001 and *****p* < 0.0001 were considered to be statistically significant.

References

1 Chu, X. *et al.* Sterol regulatory element-binding protein-1c mediates increase of postprandial stearic acid, a potential target for improving insulin resistance, in hyperlipidemia. *Diabetes*. **62**, 561-571 (2013).

2 Liu, Y. *et al.* IL-35 mitigates murine acute graft-versus-host disease with retention of graft-versus-leukemia effects. *Leukemia*. **29**, 939-946 (2015).

3 Cooke, K. R. *et al.* An experimental model of idiopathic pneumonia syndrome after bone marrow transplantation: I. The roles of minor H antigens and endotoxin. *Blood*. **88**, 3230-3239 (1996).

4 Rakoff-Nahoum, S., Paglino, J., Eslami-Varzaneh, F., Edberg, S. & Medzhitov, R. Recognition of commensal microflora by toll-like receptors is required for intestinal homeostasis. *Cell*. **118**, 229-241 (2004).

5 Chen, G. Y., Shaw, M. H., Redondo, G. & Nunez, G. The innate immune receptor Nod1 protects the intestine from inflammation-induced tumorigenesis. *Cancer Res*. **68**, 10060-10067 (2008).

6 Cai, Y. *et al.* Adoptively transferred donor IL-17-producing CD4(+) T cells augment, but IL-17 alleviates, acute graft-versus-host disease. *Cell Mol Immunol*. **15**, 233-245 (2018).

7 Eldar, A., Shapiro, O., Bejerano, Y. & Bercovier, H. Vaccination with whole-cell vaccine and bacterial protein extract protects tilapia against Streptococcus difficile meningoencephalitis. *Vaccine*. **13**, 867-870 (1995).

8 Cekanaviciute, E. *et al.* Gut bacteria from multiple sclerosis patients modulate human T cells and exacerbate symptoms in mouse models. *Proc Natl Acad Sci U S A*. **114**, 10713-10718 (2017).

9 Kaplan, D. H. *et al.* Target antigens determine graft-versus-host disease phenotype. *J Immunol*. **173**, 5467-5475 (2004).

10 Polchert, D. *et al.* IFN-gamma activation of mesenchymal stem cells for treatment and prevention of graft versus host disease. *Eur J Immunol*. **38**, 1745-1755 (2008).

11 Yang, B. *et al.* Monitoring tyrosine kinase inhibitor therapeutic responses with a panel of metabolic biomarkers in chronic myeloid leukemia patients. *Cancer Sci*. **109**, 777-784 (2018).

Figure. S1.

**Figure S1. High stearic acid diet (HSAD), but not high palmitic acid diet (HPAD), aggravates murine aGVHD and altered the levels of serum lipids.** (a-c) Survival, body weight, and clinical aGVHD score of HPAD and Normal Diet (ND) recipients after allo-BMT. (d-e) Body weight and clinical aGVHD score of HSAD and ND recipients. (f) Levels of serum lipids, including Triglyceride (TG), Total cholesterol (T-CHO), HDL cholesterol (HDL-c), and LDL cholesterol (LDL-c), which were assayed determined in the serum of HSAD and ND mice in 0,1,2,3,4 weeks post diet administration. n = 5-6 for each time point in each group. Survival curves were compared using log rank Mantel-Cox curve comparison, two-tailed Mann-Whitney U test for body weight and clinical aGVHD score. For f, two-way ANOVA for multiple comparisons. Error bars, mean ± SEM. **p*<0.05, ***p*<0.01, ****p*<0.001, *****p*<0.0001.

Figure. S2.

**Figure S2. The effect of HSAD on murine aGVHD.** (a-c) Lethally irradiated B6 recipients were reconstituted with BALB/c BM cells and splenocytes. Survival, body weight and clinical aGVHD score were monitored. (d-f) Survival, body weight and clinical aGVHD score of BALB/c recipient mice received allo-BMT from either HSAD B6 or ND B6 donors. Survival curves were compared using log rank Mantel-Cox curve comparison. Two-tailed Mann-Whitney U test for body weight and clinical aGVHD score. Error bars, mean ± SEM. **p*<0.05, ***p*<0.01, ****p*<0.001, *****p*<0.0001.

Figure. S3.

**Figure S3. Gut microbiota is involved in HSAD-induced severe aGVHD.** (a) Body weight and (b) clinical aGVHD score of ND and HSAD mice after allo-BMT treated with or without antibiotics in drinking water for 4 weeks. (c) Body weight and (d) clinical aGVHD score of ND and HSAD mice orally gavaged with stool suspensions every other day with feces from HSAD and ND group for 4 weeks. (e) Comparison of alpha diversity of gut microbiota among ND mice and HSAD mice using Shannon’s index (*p*=0.0002). (f) Bacterial beta diversity, as measured at the genus level using the principal coordinates analysis (PCoA) of the Bray-Curtis dissimilarity metric among samples among samples of the two groups. Results in a-d were analyzed with two-tailed Mann-Whitney U test. For e and f, n = 9 per group. *P* values were determined by unpaired two-tailed Student’s t-test. Data are presented as mean ± SEM; **p* < 0.05, ***p* < 0.01, ****p* < 0.001 or *****p*<0.0001.

Figure. S4.

**Figure. S4. HSAD upregulates *A.muciniphila* abundance and *A.muciniphila* supplementation aggravates aGVHD in ND mice.** (a-b) Body weight and clinical aGVHD score after allo-BMT of ND recipients treated with *A. muciniphila* and HSAD recipients treated with *B.fragilis* compared to PBS vehicle. Two-tailed Mann-Whitney U test for body weight and clinical aGVHD score. (c-d) Expression of *A.muciniphila* and *B.fragilis* were assessed through qPCR in fecal from ND+PBS, ND+*A.muciniphila*, HSAD+PBS and HSAD+*B.fragilis* mice (n=6-9 per group). Data were plotted as relative quantification normalized to the levels of β-actin. Significance between every two groups was calculated using Mann-Whitney U test. **p*<0.05, ***p*<0.01, ****p*<0.001, *****p*<0.0001, Error bars, mean ± SEM.

Figure. S5.

**Figure S5. HSAD and *A. muciniphila* aggravates aGVHD through altering overall fecal metabolism, especially the metabolite acetate.** (a) Score plot of partial least square discriminant analysis (PLS-DA) was used to determine the global metabolic profiles of HSAD and ND groups (R^2^X = 0.631, R^2^Y= 0.796, Q^2^ (cum) = 0.661). (b) Heatmap representation of the significant changes in fecal metabolites detected by GC/MS between ND and HSAD mice, after Student’s t-test, after hierarchical clustering. For a and b, n=8 per group. (c) Abundance of isobutyric acid, isovaleric acid, valeric acid, and caproic acid of bacterial culture supernatant and no-bacteria control (n=6 per group). (d) Survival of ND recipients treated with vehicle (PBS) or acetate (100 mg kg ^–1^) and HSAD recipients treated with vehicle (PBS) after allo-BMT. *P* values were determined by unpaired two-tailed Student’s t-test. Survival curves were compared using log rank Mantel-Cox curve comparison. Error bars indicate SEM. **p*<0.05, ***p*<0.01, ****p*<0.001, *****p*<0.0001.

Figure. S6.

**Figure S6. HSAD promotes T cells function *in vivo* and mainly depended on CD4^+^ T cells.** Representative flow cytometry plots and quantification of (a) activated CD4^+^ and CD8^+^ T cells, (b) effector, memory CD4^+^ and CD8^+^ T cells from spleen of HSAD and ND recipients. **p*<0.05, ***p*<0.01, ****p*<0.001, *****p*<0.0001, *p* values were determined by unpaired two-tailed Student’s t-test and data are presented as mean ± SEM.

Figure. S7.

**Figure S7. CD4^+^ T cells and its cytokines play an essential role in HSAD-mediated severe aGVHD.** (a-b) Body weight and clinical aGVHD score after allo-BMT for HSAD recipients receiving donor cells in the absence of CD4^+^ T cells or CD8^+^ T cells. Statistics were computed with two-tailed Mann-Whitney U test. (c) Representative flow cytometry plots of donor IL-17A^+^ Th17 and IFNγ^+^ Th1 lymphocytes in the spleen within the CD4^+^ population in ND, HSAD and antibiotic-treated HSAD (HSAD-Abx) recipients (n= 4-5 per group). (d) Representative flow cytometry plots of IL-17A^+^ Th17 and IFNγ^+^ Th1 lymphocytes in the spleen of ND, HSAD and ND mice receiving *A. muciniphila* (ND+ *A. muciniphila*) recipients (n = 4-5 per group). (e) Mean fluorescence intensity (MFI) of IFN-γ, IL-17A, and IL-17F from HSAD and ND mice serum samples on day 7 after allo-BMT (n = 6 per group). *P* values were determined by unpaired two-tailed Student’s t-test. **p*<0.05, ***p*<0.01, ****p*<0.001, *****p*<0.0001, Error bars, mean ± SEM.

Figure. S8.

**Figure S8. Increased Th17 and Th1 cells in HSAD mice contribute to aGVHD severity.** Body weight and clinical aGVHD score after allo-BMT of HSAD and ND recipients treated with WT B6 donors and (a) IL-17A ^−/−^ B6 donors, (b) IL-17F ^−/−^ B6 donors, or (c) IFN-γ ^−/−^ B6 donors. **p*<0.05, ***p*<0.01, ****p*<0.001, *****p*<0.0001, two-tailed Mann-Whitney U test for body weight and clinical aGVHD score. Error bars, mean ± SEM.

Figure. S9.

**Figure S9. Stearic acid (SA) promotes growth of *A.muciniphila* *in vitro* and *A.muciniphila* shows promotion effect on Th17 and Th1 cells.** (a) *A.muciniphila* was cultured in BHI+SA (stearic acid) medium with different concentrations (0, 0.1, and 1 mM, n=2 per group). Statistics in were determined by unpaired two-tailed Student’s t test with one-side relative to 0mM control. (b) Quantification of Th17 and Th1 lymphocytes under treatment of no-bacteria control or *A.muciniphila* co-cultured with SA medium in different concentrations (0, 0.1, and 1 mM). n=2 per group. Statistics were computed with the unpaired two-tailed Student’s t test. (c) The percentage of CD4^+^ IL-17A^+^ Th17 and CD4^+^ IFNγ^+^ Th1 lymphocytes from the supernatant of cultured naive CD4^+^ T cells treated with various concentrations of acetate. *P* values were determined by one-way ANOVA or Kruskal-Wallis test followed by Dunnett’s post hoc analysis with one-side relative to vehicle or 0mM control. **p*<0.05, ***p*<0.01, ****p*<0.001, *****p*<0.0001, Error bars, mean ± SEM.

Figure. S10.

**Figure S10. *A. muciniphila* promotes serum levels of IL-17A and IFN-γ.** (a) Serum levels of IFN-γ, and IL-17A for patients with non-aGVHD (n = 6) and aGVHD (n = 11). Correlations between the concentration of *A. muciniphila* in the fecal and (b) IL-17A in the serum or (c) IFN-γ in the serum, as determined by Pearson’s rank test. For a, *p* values were determined by two-tailed Mann-Whitney U test. Data represent one of three independent experiments with similar findings. Data are presented as mean ± SEM; **p* < 0.05, ***p* < 0.01, ****p* < 0.001 or *****p*<0.0001.

Table S1.

**Supplementary Table 1. Clinical characteristics of 25 allo-BMT recipients.**

| Characteristic | non-aGVHD | aGVHD |  |
| --- | --- | --- | --- |
| Number of all patients | 13 | 12 |  |
| Gender (Male/ Female) | 9/4 | 7/5 |  |
| Mean age | 32.9 (8-56) | 33 (13-60) |  |
| Primary malignancy | ALL, 7 (53.8%); AML, 4 (30.8%); MDS, 1 (7.7%); MAL, 1 (7.7%) | ALL, 4 (33.3%); AML, 4 (33.3%); MDS, 2 (16.8%); MAL, 1 (8.3%); AA, 1 (8.3%) |  |
| Disease status at allo-BMT | CR, 13 (100%) | CR, 11 (91.7%); NR, 1 (8.3%) |  |
| aGVHD grade | 0 grade, 13 (100%) | 1-2 grade, 6 (50%);  3-4 grade, 6 (50%) |  |
| Graft source | PSC, 4 (30.8%); BM + PSC, 2 (15.4%); PSC + CB, 7 (53.8%) | PSC, 3 (25%); BM, 2 (16.7%); BM + PSC, 3 (25%); BM + CB, 1 (8.3%); PSC + CB, 2 (16.7%); BM + PSC + CB, 1 (8.3%) |  |
| Conditioning intensity | reduced intensity myeloablative, 13 (100%) | Standard intensity myeloablative, 1 (8.3%); reduced intensity myeloablative, 10 (83.4%); nonmyeloablative, 1 (8.3%) |  |
| HLA match | HLA match, 2 (15.4%); HLA mismatch, 11 (84.6%) | HLA match, 2 (16.7%); HLA mismatch, 10 (83.3%) |  |
| ABO blood type | match, 6 (46.2%); mismatch, 7 (53.8%) | match, 5 (41.7%); mismatch, 7 (58.3%) |  |
| Donor/recipient sex | match, 7 (53.8%); mismatch, 6 (46.2%) | match, 7 (58.3%); mismatch, 5 (41.7%) |  |
| Mean MNC (*10^8^/kg) | 8.60 (3.52-15.5) | 9.66 (4.76-12.1) |  |
| Mean CD34 ^+^ cells (*10^6^/kg) | 3.52 (1.97-6.57) | 3.85 (1.88-5.96) |  |
| Broad-spectrum antibiotics | for neutropenic fever, 13 (100%) | for aGVHD, 12 (100%) |  |

ALL, acute lymphoblastic leukemia; AML, acute myelogenous leukemia; MDS, myelodysplastic syndromes; MAL, mixed acute leukemia; AA, aplastic anemia; CR, complete remission; NR, non-remission; BM, bone marrow; PSC, peripheral stem cells; CB, cord blood; HLA, human lymphocyte antigen, Donor-patient pairs were considered matched if all 10 HLA-A, -B, -C, -DRB1, and -DQB1 alleles matched for related and unrelated marrow; MNC, mononuclear cells.
